# An assessment of stigma and human right violations among men who have sex with men in Abuja, Nigeria

**DOI:** 10.1186/s12914-019-0190-x

**Published:** 2019-03-05

**Authors:** Susanne Strömdahl, Abimbola Onigbanjo Williams, Bede Eziefule, Godwin Emmanuel, Stella Iwuagwu, Oliver Anene, Ifeanyi Orazulike, Chris Beyrer, Stefan Baral

**Affiliations:** 1 Centre for Public Health and Human Rights, Department of Epidemiology, Johns Hopkins Bloomberg School of Public Health, Baltimore, USA; 2 Department of Medical Sciences, Section of Infectious Diseases, Uppsala University, Uppsala, Sweden; 3 Centre for the Right to Health Nigeria (CRH), Abuja, Nigeria; 4 Alliance Rights Nigeria (ARN), Abuja, Nigeria

**Keywords:** Men who have sex with men, Stigma, Human rights violations, HIV, STI

## Abstract

**Background:**

There have been several barriers in effectively engaging men who have sex with men for STI/HIV prevention and treatment programming in Nigeria including social stigma, policies, and laws criminalizing same-sex practices. The objective of this study was to describe the human rights context for MSM in Abuja and characterize factors associated with having had a genital ulcer disease in the previous 12 months, a health outcome associated with increased risk of HIV acquisition and transmission.

**Methods:**

A convenience sample of 297 men reporting ever having had anal intercourse with another man participated in the study in 2008. A structured survey instrument including sexual risk behaviour for STI/HIV, disclosure of sexual orientation, perceived and enacted human rights violations were performed. Descriptive and inferential data analyses were conducted using Stata11 software.

**Results:**

36% reported having been discriminated due to sexual orientation and 17% reported being afraid to walk the streets of their community. Enacted rights violations included 41% having been blackmailed, 36% been beaten, 13% been denied housing, and 11% been jailed due to sexual orientation. Having been blackmailed due to sexual orientation (aOR 3.40, 95%CI: 1.35–8.56) was significantly associated with reporting having had a genital ulcer in the last 12 months. Having been beaten due to sexual orientation (aOR 2.36, 95%CI:0.96–5.82) was moderately significantly associated with reporting having had a genital ulcer in the last 12 months.

**Conclusions:**

High levels of experienced stigma, discrimination and human rights violations among MSM in Abuja was reported, constituting structural risks that are linked to sexual risk behaviour for STI/HIV. Given data on the high prevalence and incidence of HIV among MSM in Abuja, these findings reinforce the need for structural interventions to mediate access to STI/HIV prevention and treatment services.

## Background

Studies have consistently demonstrated across diverse economic and geographical contexts that stigma and human rights violations disproportionately affect gay men and other men who have sex with men (MSM). Perceived and enacted stigma have been found to be associated with sexual high risk behaviour for HIV and decreased rates of HIV testing among MSM ranging from Southern Africa to Eastern Asia to Western Europe [1–4].

Same sex practices are criminalized under federal sodomy laws and punishable by lengthy prison sentences and even death by stoning in twelve of Nigeria’s 36 States [5]. The Same Sex Marriage Prohibition Act, was signed on Jan 13th 2014 further criminalizing same-sex relationships, public display of same-sex amorous relationships and membership of gay clubs and organizations with penalties of up to 14 years of incarceration [6]. Following the approval of the bill, there have been reported increases in the arrests of gay men and other MSM in Nigeria [7].

HIV prevalence in Nigeria has been estimated to be 3.1% in 2016 [8]. Due to Nigeria’s large population of 186.5 million inhabitants this translates to that the country holds about 10% of the global burden of HIV [9]. The 2014 Integrated Biological and Behavioural Surveillance Study (IBBSS) among MSM estimated HIV prevalence to be 22.9%, an increase compared to the IBBSS 2010 that reported a HIV prevalence of 17.2% [10, 11]. These estimates are about five times higher than the HIV prevalence in the general population [12, 13].

A prospective cohort study of MSM, the TRUST cohort, recruited 862 MSM in Nigeria between 2013 and 16 and reported a 55% prevalence of HIV. Chlamydia was diagnosed among 17% in Abuja and 18% in Lagos and gonorrhoea among 19% in Abuja and 26% in Lagos indicating a high burden of sexually transmitted infections (STI) [14]. Incident STI was found to be associated with HIV infection [15].

Sexual risk behaviours for HIV and STIs overlap, with the most efficient mode of HIV transmission through sexual contact being condomless anal sex with a serodiscordant viremic person [16]. Genital ulcerative diseases (GUD) are caused by STIs and introduces a breakage in the genital mucosa and genital bleeding which increases the acquisition risk for HIV [17, 18]. GUD further enhances HIV transmission from an HIV-infected individual by increasing genital HIV shedding [19–22].

Health care facilities play an important role for successful antiretroviral treatment (ART) to achieve viral suppression and thereby also limiting onwards transmission, known as Treatment as Prevention (TasP). The TRUST MSM cohort in Nigeria reported that disclosure of being MSM to a health care provider was associated with treatment adherence and HIV viral load suppression at 6 month follow up [23]. After the Same Sex Marriage Prohibition Act was passed in 2014 a substantial part of the TRUST cohort was lost to follow up. Those MSM remaining in the cohort reported a significant increase in fear of seeking health care [24]. Fear of seeking health care has been reported in other African contexts as well, 17% among MSM in Malawi, Namibia and Botswana reported fear of seeking health care. Having experienced discrimination due to sexual orientation was found to be associated with fear of seeking health care and was more common among those on ART [1].

Considering the high HIV incidence and prevalence reported among MSM in Nigeria, Pre Exposure Prophylaxis for HIV (PrEP) hold promise to help curb the epidemic [25]. However PrEP is dependent on that MSM are willing to visit health care facilities for regular HIV/STI testing and follow-up. Thereby fear of seeking health care and fear of disclosing as MSM may act as a barrier to PrEP uptake. These data points towards that stigma and criminalization play an important role in the HIV/STI epidemic among MSM in Nigeria, acting as a structural barrier to HIV/STI prevention and treatment.

The objective of this study was to describe the human rights contexts for MSM in Abuja in 2008, before the Same Sex Marriage Prohibition Act was passed. In addition, to characterize factors associated with reporting having had a GUD in the previous 12 months, a health outcome that is associated with HIV acquisition and transmission risk. A modified social ecological model (MSEM) was used to assess HIV risk [26]. This study was implemented in partnership between the Centre for Right to Health, a Non Governmental Organization working for access to health care for vulnerable groups, and Alliance Rights Nigeria, a Non Governmental Organization focused on addressing the needs of MSM in Abuja.

## Methods

### Study population and accrual methods

The study method has been described in detail in the parent study (17). Briefly, the study was performed in 2008 and includes a convenience sample of 297 MSM in Abuja, Nigeria. Inclusion criteria included men aged 18–65 years old, living in Abuja who self-reported ever having had anal intercourse with another man. A structured survey face to face with a trained peer in a safe and private environment to ensure confidentiality was performed. The survey included modules on socio-demographic characteristics, sexual orientation and practices, condom use during last six months, number of partners, and knowledge of HIV/STIs. A module of the survey was dedicated to disclosure of sexual orientation, stigma, discrimination, and perceived and enacted human rights abuses. The MSEM model was used to guide the data collection and analysis including different levels of HIV/STI risk contexts (29). Informed consent was obtained from all participants.

### Statistical analysis

Exploratory, descriptive and inferential data analysis was conducted using Stata 11.1 software (College Station, Texas). Descriptive statistics with chi-square and t-test were used for socio-demographic characteristics, disclosure of sexual orientation, enacted and perceived human rights violations and reported genital ulcer in past 12 months. The predictor variables were dichotomized and univariate binomial logistic regression and t-test were used to identify factors associated with reported genital ulcer in the last 12 months.

Fifthteen variables representing multi-levels of HIV/STI risk in the MSEM model were tested for association with self-reported genital ulcer in the last 12 months [26]. Backward elimination with a *p*-value set to 0.1, locking the variables age and tertiary education in the model due to being hypothesized confounder in the conceptual framework, was used to determine which variables to include in the multivariate model. The Hosmer-Lemeshow test was performed to assure goodness of fit [27]. The likelihood ratio test was used to test for interaction between the predicting variables; blackmailed due to sexual orientation and having been beaten due to sexual orientation. The stability of the model was confirmed by Akaike information criterion (AIC) values [28]. Variables with a (*p* < 0.05) were considered significantly associated with the outcome and variables with a (*p* < 0.1) were considered moderately significantly associated with the outcome; self-reported genital ulcer in the previous 12 months.

## Results

### Socio-demographic characteristics of the study population

Table 1 describes the characteristics of the 297 MSM who participated in the study. Almost all MSM participating in this study, 95% (*n* = 282/297) had a secondary school or higher educational level. However, only 59% (*n* = 175/297) were employed. Age of participants ranged between 18 and 45 years old with a median of 26. All three large ethnic groups in Nigeria were represented; Igbo 37%(*n* = 107/289), Yoruba 18%(*n* = 53/289), and Hausa 15%(*n* = 44/289). Only 15%(*n* = 43/294) reported belonging to a MSM community-based association. A fifth of the study population,19% (*n* = 54/289), reported having had a genital ulcer in the last 12 months.

**Table 1:** Socio-demographic Characteristics of MSM in Abuja

Characteristic	Percentage	Absolute #
Age (*n* = 295)	Mean 26 Median 26.05	Range 18–45
Education		
Primary school	5.05%	15/297
Secondary school	49.83%	148/297
Tertiary or vocational school	45.12%	134/297
Employment	58.92%	175/297
Religion		
Christian	78.17%	222/283
Muslim	17.25%	49/283
Traditional	0.7%	2/283
No religion	3.53%	10/283
Ethnic group		
Yoruba	18.34%	53/289
Igbo	37.02%	107/289
Hausa	15.22%	44/289
Other	29.41%	85/289
Sexual self identification		
heterosexual/straight	1.34%	4/299
homosexual/gay	35.45%	106/299
bisexual	63.21%	189/299
transgender	0%	0/299
Genital ulcer in past 12 months	18.7%	54/289

### Disclosure of sexual orientation

15% (n = 44/288), reported that they were open regarding their sexual orientation in their community. Somewhat less, 11% (*n* = 32/293) reported that their community was aware of their sexual orientation. One fourth, 25% (*n* = 72/293) had disclosed their sexual orientation to their immediate family. About one third, 28% (*n* = 84/297) had disclosed their sexual orientation to a health worker. Further details are described in Table 2.

**Table 2:** Disclosure of sexual orientation among MSM in Abuj

Characteristic	Percentage	Absolute #
Open to community	15.28%	44/288
Community aware of sexual orientation	10.92%	32/293
Disclosed sexual orientation to immediate family	24.57%	72/293
Disclosed sexual orientation to extended family	12.85%	37/288
Disclosed sexual orientation to health worker	28.28%	84/297
Disclosed sexual orientation to religious leader	18.06%	52/288

### Stigma, discrimination and human rights violations

As described in Table 3, more than a third, 36% (*n* = 102/284) reported having been discriminated due to their sexual orientation. Close to half, 41% (*n* = 116/286) had been blackmailed due to their sexual orientation. Almost one third, 28% (*n* = 81/289) had been arrested and a tenth,11% (*n* = 31/287) had been arrested due to their sexual orientation. 36% (101/278) had been beaten due to their sexual orientation, 7% (*n* = 20/278) by a family member and 8% (*n* = 21/278) by a sexual partner. 3% (*n* = 9/278) reported having been beaten by police or government officials due to their sexual orientation. About a fifth, 18% (*n* = 51/281) reported having been raped of which 24% (*n* = 12/51) reported rape to authorities/police. Only 1% (n = 2/295) had been denied health care due to their sexual orientation.

**Table 3:** Human rights contexts

Characteristic	Percentage	Absolute #
Discriminated due to sexual orientation	35.92%	102/284
Denied housing due to sexual orientation	12.63%	36/285
Denied health care due to sexual orientation	0.68%	2/295
Discriminated in place of worship	25.42%	15/59
Afraid to walk in their community	15.27%	42/275
Blackmailed due to sexual orientation	40.56%	116/286
Have been arrested/jailed	28.20%	81/289
Have been arrested/jailed due to sexual orientation	10.80%	31/287
Access to condoms in jail	1.45%	1/69
Beaten due to sexual orientation	36.33%	101/278
Beaten by police/government officials due to sexual orientation	3.24%	9/278
Beaten by family member due to sexual orientation	7.19%	20/278
Beaten by sexual partner	7.55%	21/278
Been raped	18.15%	51/281
Reported rape	23.53%	12/51
Prosecuted rape	2.63%	1/38

### Associations with having had a genital ulcer in the last 12 months

In the univariate analysis described in Table 4, the following variables were found to be significantly associated with having had a GUD in the previous 12 months; having received money/gifts for sex (OR 2.67, 95%CI: 1.30–5.48), been denied housing due to sexual orientation (OR 2.98, 95%CI: 1.39–6.41), been arrested (2.13, 95%CI: 1.14–3.96), been arrested due to sexual orientation (OR 3.88, 95%CI: 1.75–8.60), beaten due to sexual orientation (OR 3.68, 95%CI: 1.96–6.91), blackmailed due to sexual orientation (OR 3.54, 95%CI 1.89–6.60), and been raped (OR 2.30, 95%CI: 1.15–4.58). The following variables were found to be significantly inversely associated with having had a GUD in the previous 12 months; tertiary schooling (OR 0.29, 95%CI:0.15–0.58), knowledge that HIV/STIs can be transmitted through unprotected anal intercourse (OR 0.34, 95%CI: 0.13–0.89). In the multivariate analysis, having been blackmailed due to sexual orientation (aOR 3.40, 95%CI: 1.35–8.56) was significantly associated with having had a GUD in the previous 12 months. Having been beaten due to sexual orientation (aOR 2.36, 95%CI:0.96–5.82) was moderately significantly associated with having had a GUD in the previous 12 months.

**Table 4:** Associations of genital ulcer in previous 12 months among MSM in Abuja

Variable	Prevalence (Absolute#)	Univariate	Multivariate
Age > 26	45%(*n* = 134/295)	1.53(0.84–2.78)	1.12(0.56–2.24)
Tertiary education	45%(n = 134/297)	0.29(0.15–0.58) **	0.55(0.25–1.19)
Employed	59%(n = 175/297)	1.41(0.76–2.64)	
Consistent condom use with male partners	53%(*n* = 155/290)	0.65(0.36–1.19)	
Knowledge of HIV/STI transmission risk at condomless anal sex	91%(*n* = 200/220)	0.34(0.13–0.89)**	
Bisexual concurrency	66%(*n* = 179/271)	0.78(0.40–1.54)	
Ever HIV-tested	65%(*n* = 180/276)	0.56(0.30–1.07)	
Received money/gifts for sex	62%(*n* = 169/274)	2.67(1.30–5.48)**	
Denied housing due to sexual orientation	13%(*n* = 36/285)	2.98(1.39–6.41) **	
Afraid to walk the streets of their community	15%(*n* = 42/275)	1.96(1.06–3.63)	
Been arrested	28%(n = 81/289)	2.13(1.14–3.96)**	
Been arrested due to sexual orientation	11%(n = 31/287)	3.88(1.75–8.60)**	
Beaten due to sexual orientation	36%(*n* = 101/278)	3.68(1.96–6.91)**	2.36(0.96-5.82)*
Blackmailed	41%(*n* = 116/286)	3.54(1.89–6.60)**	3.40(1.35-8.56)**
Raped	18%(*n* = 51/281)	2.30(1.15–4.58)*	

*p-value < 0.05

**p-value < 0.01

## Discussion

Human rights contexts were challenging for gay men and other MSM in Nigeria in 2008. Since this study was performed same sex practices has been further criminalized in 2014 [24]. Concurrently, the evidence base of a sustained and disproportionate burden of HIV and STIs among these men is growing [11, 14, 23]. The data presented here highlight the statistically significant relationship between perceived and enacted rights violations and GUD.

The data concludes that having been blackmailed or beaten due to sexual orientation as MSM is associated with having had a GUD recently, which increases the risk of HIV transmission at condomless sex. High levels of stigma, perceived and enacted human rights violations were found. More than a third reported having been beaten due to their sexual orientation. Two fifths of participants reported having been blackmailed due to sexual orientation, supporting previous similar findings among MSM in Malawi, Botswana, Namibia and Nigeria [1, 4, 29]. Recent data suggest that fear of seeking health care and disclosing as MSM remain barriers to uptake of HIV/STI prevention and treatment in Nigeria [24].

As HIV prevalence among MSM in Abuja has been estimated to be as high as 30–50%, ART is a core component of combination HIV prevention programming for MSM [11, 14]. Structural stigma and criminalization poses a significant barrier to TasP uptake among MSM limiting the potential effect on the HIV epidemic. As reported from the TRUST cohort study there was a significant drop out among MSM participants on ARV after the passing of the Same Sex Marriage Prohibition Act in 2014 [24]. The on-going TRUST cohort and other studies are needed to explore how to overcome these barriers in settings where same sex practices are criminalized.

In addition PrEP is becoming available for MSM in some African settings, both South Africa and Kenya have approved PrEP for MSM [30]. Due to the high burden of HIV among MSM in Nigeria PrEP would be most beneficial for sero negative MSM at risk of HIV. Disclosure of same sex practices to health care providers is key to be able to do appropriate risk stratification and prescribe PrEP for those in need. To target those men in need of PrEP also holds relevance to ensure that PrEP programmes are cost-effective. Encouragingly, about a third had disclosed their sexual orientation to a health provider and only 1% of MSM had been denied health care. Similar results were reported among MSM in Malawi, Botswana and Namibia [1].

When applying the MSEM model to MSM in Nigeria, we find HIV risks at all layers including individual, network, community and public policy [26]. At the individual risk level, about a fifth of MSM participating in this study reported having had a GUD in the past 12 months. At the social network level, about a fifth had disclosed their sexual orientation to immediate family, which could provide social support for the individual. However, about a tenth had been beaten by a family member due to their sexual orientation suggesting that disclosure to family might not offer social support. The high prevalence of HIV and STIs reported in Abuja suggest a high HIV risk within the sexual network [14, 31]. At the public policy level, laws criminalizing MSM organizations create a barrier to peer HIV/STI prevention programmes and peer support programmes for adherence to TasP. Finally, a very high level of enacted human rights violations were found in this study, stressing that factors at the community level poses a barrier for MSM to access needed HIV/STI services. Thereby we also find structural stigma towards MSM embedded in all ecological layers, including the individual, social network, community and public policy level. A recent study among MSM in Togo stressed further that the network, community and policy level factors was associated with HIV infection [32].

The data reported here implicates that structural stigma, perceived and enacted human rights violations are barriers to uptake of health services among MSM. As well as acting as barriers to implement improved public health programming for this population. Future comprehensive HIV preventions for MSM should include components on human rights and stigma for the individual MSM, at the social network level through peer support groups, the community level through awareness campaigns, and to aspire to impact the public policy level and lead to a movement towards equal rights [33]. Further studies can contribute by making data on HIV, stigma and human rights available to highlight the current situation among MSM in Nigeria.

The recruitment for this study was performed as a convenience sample in 2008, generalizability is thereby limited. Some years have passed since these data were collected and the legal climate in Nigeria has changed with harsher criminalization of same sex practices. Thereby these data highlight the human rights context among MSM in Abuja prior to these legal changes. External validity is further limited as this study was performed in Abuja, an urban centre in Nigeria. The study design is cross-sectional and thereby causality cannot be detected. Self-reported data includes a risk of reporting- and social desirability- bias. The study was performed in collaboration with a MSM community and employed members of the MSM community at all stages of the study including design, instrument development and implementation to ensure safety of the participants and to minimize social desirability bias.

## Conclusion

High levels of stigma, perceived and enacted human rights violations were reported among MSM in Abuja in 2008. Having been blackmailed or beaten due to sexual orientation was found to be associated with having had a GUD recently. Since 2008 the legal climate in Nigeria has changed with the passing of more punitive laws further criminalizing same sex practices. In addition, the HIV/STI epidemics remain at high prevalence levels among MSM. Fear of disclosing as MSM and fear of seeking health care construct barriers for uptake of HIV/STI prevention including PrEP and universal coverage of ART among MSM at risk for and living with HIV, respectively. While efficacy data support the need for ART-based strategies at scale, studying implementation models of effective scaling is required given the sustained and increasing structural challenges to service provision for MSM in Nigeria.

## Abbreviations

ART: Antiretroviral treatment
GUD: Genital ulcer disease
IBBSS: Integrated biological and behavioural surveillance study
MSEM: Modified social ecological model
MSM: Men who have sex with men
PrEP: Pre exposure prophylaxis for HIV
STI: Sexually transmitted infections
TasP: Treatment as prevention
